# Chemical and Bio Sensing Using Graphene-Enhanced Raman Spectroscopy

**DOI:** 10.3390/nano9040516

**Published:** 2019-04-02

**Authors:** Alexander Silver, Hikari Kitadai, He Liu, Tomotaroh Granzier-Nakajima, Mauricio Terrones, Xi Ling, Shengxi Huang

**Affiliations:** 1 Department of Electrical Engineering, The Pennsylvania State University, University Park, PA 16802, USA; ajs721@psu.edu; 2 Department of Chemistry, Boston University, Boston, MA 02215, USA; hk91@bu.edu (H.K.); xiling@bu.edu (X.L.); 3 Department of Chemistry, The Pennsylvania State University, University Park, PA 16802, USA; hbl5085@psu.edu (H.L.); mut11@psu.edu (M.T.); 4 Department of Physics, The Pennsylvania State University, University Park, PA 16802, USA; txg210@psu.edu; 5 Department of Materials Science and Engineering and Center for 2-Dimensional and Layered Materials, The Pennsylvania State University, University Park, PA 16802, USA; 6 Division of Materials Science and Engineering, Boston University, Boston, MA 02215, USA; 7 The Photonics Center, Boston University, Boston, MA 02215, USA

**Keywords:** 2D materials, biochemical sensing, graphene-mediated surface enhanced Raman spectroscopy, chemical mechanism, nanocomposite

## Abstract

Graphene is a two-dimensional (2D) material consisting of a single sheet of sp^2^ hybridized carbon atoms laced in a hexagonal lattice, with potentially wide usage as a Raman enhancement substrate, also termed graphene-enhanced Raman scattering (GERS), making it ideal for sensing applications. GERS improves upon traditional surface-enhanced Raman scattering (SERS), combining its single-molecule sensitivity and spectral fingerprinting of molecules, and graphene’s simple processing and superior uniformity. This enables fast and highly sensitive detection of a wide variety of analytes. Accordingly, GERS has been investigated for a wide variety of sensing applications, including chemical- and bio-sensing. As a derivative of GERS, the use of two-dimensional materials other than graphene for Raman enhancement has emerged, which possess remarkably interesting properties and potential wider applications in combination with GERS. In this review, we first introduce various types of 2D materials, including graphene, MoS_2_, doped graphene, their properties, and synthesis. Then, we describe the principles of GERS and comprehensively explain how the GERS enhancement factors are influenced by molecular and 2D material properties. In the last section, we discuss the application of GERS in chemical- and bio-sensing, and the prospects of such a novel sensing method.

## 1. Graphene and Graphene-Like 2D Materials

The experimental isolation of graphene, a single layer of carbon atoms, opened the door to the world of two-dimensional (2D) materials by demonstrating the experimental existence and physical stability of atomically thin layers [1]. The extremely large ratios of surface area to volume of 2D materials make them sensitive to various types of interactions on their surface, a foundation for sensing devices.

**Figure 1:**
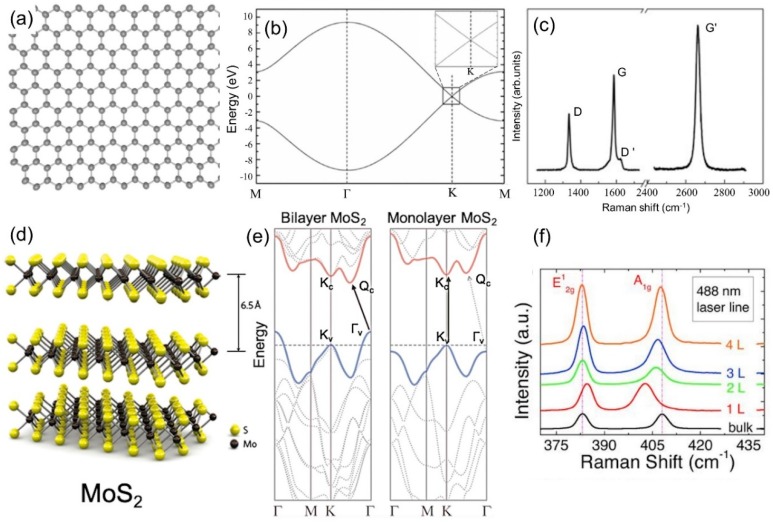
(**a**) Atomic model of graphene. (**b**) Electronic band structure of graphene. The inset is an enlargement of the square region around the K point. Reproduced from [4] with permission (American Physical Society, 2006). (**c**) Raman spectra of graphene displaying the D, G, D′, and G′ (2D) bands. Reproduced from [5] with permission (Elsevier, 2009). (**d**) Crystal structure of MoS_2_. Reproduced from [6] with permission (American Chemical Society, 2010). (**e**) Indirect-direct band gap crossover. Reproduced from [6] with permission. (**f**) Layer dependence Raman spectra of MoS_2_. Reproduced from [7] with permission (Wiley-VCH, 2012).

Graphene is made of sp^2^-hybridized carbon atoms arranged in two intersecting triangular lattices that combine to form a honeycomb pattern (Figure 1a). Compared to its bulk counterpart, graphite, the band structure of graphene is unique: It exhibits Dirac cones at the K points in the Brillouin zone (Figure 1b). This causes the low-energy electron excitations to behave as massless Dirac fermions, which exhibit unconventional phenomena, including the anomalous integer quantum Hall effect [2] and Klein tunneling [3].

Graphite is formed by stacking graphene layers that are held together by van der Waals forces. This weak interlayer coupling force means it is easy to isolate individual graphene layers. The first methods used to isolate graphene involved mechanical exfoliation using “scotch tape” [1]. Since then, other methods have been developed to produce graphene, including chemical vapor deposition (CVD), which allows for the roll-to-roll continuous production of highly crystalline graphene on Cu foils [8]. CVD is often carried out using methane gas (CH_4_) as a carbon-containing precursor, and Cu substrates that act as a dehydrogenating and surface-growth limiting catalyst for a high-quality monolayer coverage [9].

CVD also allows the introduction of heteroatoms within the graphene lattice, known as substitutional doping, to tune the physico-chemical properties of graphene. Incorporating precursors, such as ammonia (NH_3_), in CVD results in the nitrogen doping of graphene [10,11,12]. Incorporating nitrogen into graphene lattices can tune the electronic properties by shifting the Fermi level and in some cases, opening a band gap [13]; it can also enhance the catalytic and sensing properties by changing the local charge distribution around the dopants [14,15,16,17,18].

Boron and silicon have also been used to dope graphene and enhance its sensing performance [16,19]. Lv et al. found that boron p-dopes graphene and it is frequently found as part of a croissant-shaped feature consisting of boron-carbon trimers reconstructing vacancies within the lattice [16]. When used to make a gas sensing device, boron-doped graphene achieved sensitivities down to 95 parts per trillion (ppt) and 60 parts per billion (ppb) for NO_2_ and NH_3_, respectively [19]. In a separate paper, Lv et al. doped graphene with Si, which was shown to enhance the Raman signal of adsorbed dye molecules [19]. For certain molecules, this graphene-enhanced Raman scattering (GERS) effect is stronger for silicon-doped graphene than pristine or nitrogen-doped graphene.

Semiconducting layered transition metal dichalcogenides (TMDs), such as molybdenum disulfide (MoS_2_), have attracted considerable research attention due to a series of novel physical properties when thinned down to few-layers or a single monolayer [20,21,22]. Typically, each layer of TMD material has a thickness of 6–7 Å, which consists of a hexagonally packed layer of transition metal atoms sandwiched between two layers of chalcogen atoms (Figure 1d) [23]. In contrast to the gapless graphene, TMDs (e.g., MoS_2_ and WS_2_) have finite bandgaps, which undergo a transition from the indirect bandgap in their multilayer form, to a direct bandgap in their monolayer limit [24,25], as shown in Figure 1e. Similar to graphene, mechanical exfoliation, chemical exfoliation (e.g., lithium intercalation [26]), and CVD (e.g., with sulfur powder and MoO_3_ film [27]) can also be used to synthesize high quality monolayers or few-layer crystals.

Raman spectroscopy (named after the Indian physicist, Sir C. V. Raman) is a spectroscopic technique used to observe vibrational, rotational, and other low-frequency modes in a system [28]. It relies on inelastic scattering of monochromatic light, usually from a laser source. The laser light interacts with molecular vibrations, phonons, or other excitations, resulting in the energy of the laser photons being shifted up or down. The shift in energy gives information about the vibrational modes in the system. Raman spectroscopy is a useful tool used to characterize graphene and TMDs, as it can indicate the layer number and defect density in a non-destructive manner. For graphene, there are two main bands in the Raman spectrum (Figure 1c), the G band (~1580 cm^−1^) and the G’ band (~2700 cm^−1^), also referred to as the 2D band. In defective graphene, there is also a defect active D band (~1350 cm^−1^) at about half the frequency of the G’ band. The G band is a first order Raman scattering process coming from two degenerate phonon modes at the Γ point. The D band is a second order Raman mode consisting of an absorption of a photon by an electron at the K point, inelastic scattering by a phonon to the K’ point, and then elastic scattering by a defect back to the K point followed by recombination with a hole and photon emission. The G’ band mechanism is similar to the D band, but the second elastic scattering of the electron is replaced by another inelastic scattering event off of a phonon before hole recombination and photon emission.

Raman spectroscopy of graphene is useful in that it can characterize a graphene sample non-destructively. The ratio of the G’ band to the G band as well as the shape of the G’ band was shown to indicate the layer number by Ferrari et al. [29]. In particular, they showed that the G’ band can be modeled by a single mode for monolayer graphene whereas for bilayer graphene, the G’ band splits into four components. The evolution of the D band as a function of the interdefect distance was investigated by Lucchese et al. [30]. They found that the ratio of the intensity of the D band to the G band increases for increasing interdefect distances up to approximately 5 nm and then the ratio decreases as the interdefect distance increases beyond that point.

Figure 1f shows the Raman spectra of few and monolayer MoS_2_ excited by a 488 nm laser in air. There are two main bands in the Raman spectrum (Figure 1f) for both bulk and few layer MoS_2_: The in-plane phonon mode, E’ (~382 cm^−1^), and the out of plane phonon mode, A’_1_ (~403 cm^−1^) [7]. The frequency of the E’ band decreases while the A’_1_ increases when the layer number increases. This frequency separation between the two modes can be used to distinguish the layer number of MoS_2_ [7]. The optical properties of MoS_2_ are also associated with their indirect-to-direct band gap transition from a multilayer to a monolayer (Figure 1e), featured by a strong optical absorption and luminescence emission within the visible spectral range for monolayer MoS_2_ [22].

## 2. GERS Mechanisms

### 2.1. Raman Enhancement Effect on Graphene and Beyond

The observation of a surface-enhanced Raman scattering (SERS) effect on graphene was first reported at the end of 2009 by Ling et al., where it was demonstrated that probe molecules deposited on graphene gave significantly higher Raman signals than those deposited on bare substrates [31]. The researchers evaporated sub-monolayer amounts of phthalocyanine (Pc), rhodamine 6G (R6G), protoporphyrin IX (PPP), and crystal violet (CV), on mechanically exfoliated graphene, and compared the Raman intensities with those observed on SiO_2_/Si substrates. It was found that the intensity of Pc vibrational modes was 2–17 times (depending on the vibrational mode) greater when deposited on monolayer graphene [32]. The nature of the graphene-enhanced Raman scattering (GERS) enhancement was assigned to the chemical mechanism (CM) [32].

**Figure 2:**
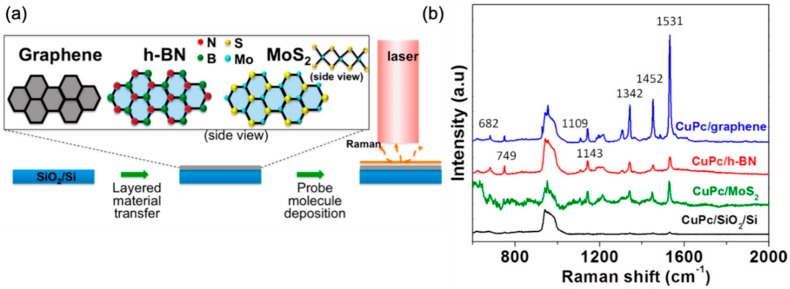
(**a**) Experimental schematics of GERS. 2D materials are mechanically exfoliated onto a SiO_2_/Si substrate. CuPc molecules are thermally evaporated under vacuum onto the 2D materials. Raman measurement is performed under a 633 nm laser excitation. (**b**) Raman spectra of CuPc on graphene (blue), h-BN (red), MoS_2_ (green), and blank SiO_2_/Si substrate (black). Reproduced from [31] with permission (American Chemical Society, 2014).

Since the discovery of GERS, many other two-dimensional layered van der Waals materials have been shown to exhibit a similar capability to enhance the Raman signals of absorbed molecules [31,33,34,35,36]. For example, Ling et al. further reported the Raman enhancement effect of thermally evaporated copper Pc (CuPc) on 2D materials with a wide range of bandgaps, from semi-metallic graphene to semiconducting MoS_2_, to insulating hexagonal-BN (Figure 2) [31]. In addition to many more vibrational modes being observed, it was found that the enhancement factor (EF) for the B_2g_ mode at 1531 cm^−1^ of CuPc was about 63 for graphene and 13 for hexagonal boron nitride (h-BN). More recently, WSe_2_ was shown to outperform the enhancement capability of graphene by 1.7 times at the 1365 cm^−1^ mode of R6G molecules [37].

**Figure 3:**
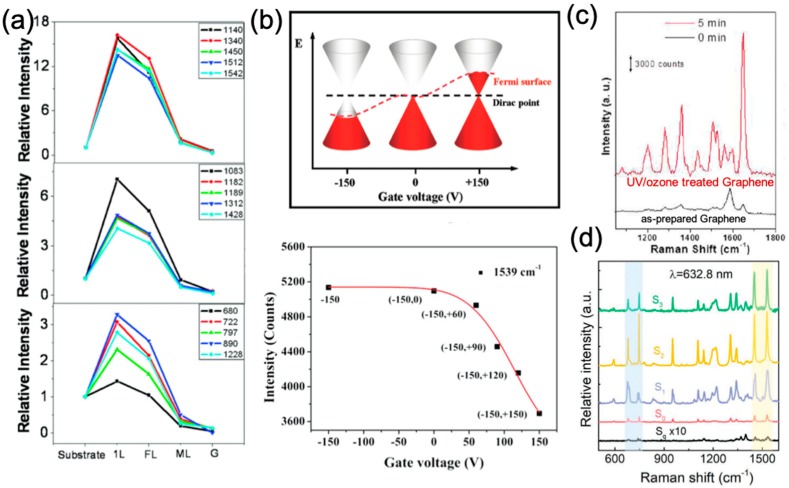
(**a**) Relative intensities of the vibrational modes of CuPc molecules. Reproduced from [32] with permission (American Chemical Society, 2010). (**b**) The effect of tuning the Fermi level of graphene on GERS. Reproduced from [45] with permission (American Chemical Society, 2011). (**c**) Enhanced Raman spectra on graphene after UV/ozone treatment. Reproduced from [46] with permission (American Chemical Society, 2011). (**d**) Effect of Se vacancies in monolayer WSe_2_ on the enhanced Raman spectra of CuPc molecules. Reproduced from [47] with permission (Wiley-VCH, 2018). Spectrum of CuPc on quartz in black (S_q_), on as-prepared WSe_2_ in red (S_0_), on WSe_2_ irradiated by an Au ion beam with fluence of S_1_ = 1 × 10^12^ ions cm^−2^ in blue; S_2_ = 1 × 10^13^ ions cm^−2^ in yellow; and S_3_ = 1 × 10^14^ ions cm^−2^ in green. Additionally, these materials are atomically flat and no covalent bond between the molecules and 2D materials is formed. Thus, they result in uniform enhancements over larger areas when compared to traditional “hot-spot”-based SERS substrates. Therefore, these 2D layered materials offer a novel and unique platform for studying the CM without interference from the EM. Moreover, the enhancements are not limited to monolayer 2D materials, but can extend to few-layers, offering flexibility when fabricating the 2D materials as Raman enhancement substrates for practical applications [48,49].

While the exact origin of this phenomenon is still under investigation, the electromagnetic mechanism (EM) is readily excluded as a possible contributor, as the surface plasmon resonance (SPR) of graphene lies in the terahertz (THz) region [38], while the excitation laser wavelengths for the Raman measurements are in the visible range. In the case of the semiconducting transition metal dichalcogenides, the surface plasmon (SP) frequency depends on the electron density, therefore, the SP originating from the conduction band are expected to lie in the infrared region, whereas the SP from the valence band would lie in the ultra-violet (UV) region [39,40,41]. Moreover, a commonly reported feature of surface enhanced Raman spectra is that the EF is dependent on the symmetry of the vibrational mode [42,43] (Figure 3a). In the CM description [44], the EFs are dictated by the chemical interactions established between the molecule and the substrate (i.e., the 2D material), where vibrational selection rules apply and vibrations with different symmetries are affected differently.

### 2.2. Dependence on Electronic Properties of 2D Materials

Benefitting from the tunability of the electronic properties of 2D materials through electric field gating, doping, or surface chemical functionalization, numerous studies have been carried out to investigate the influence of the property changes of 2D materials on the enhancement effect. In this context, Xu et al. reported that the EF is sensitive to the position of the graphene’s Fermi level (EF). As shown in Figure 3b, the Fermi level of graphene will be downshifted by applying a negative gate bias, and upshifted upon applying a positive gate bias [45]. The Raman intensity of the 1539 cm^−1^ mode of the CoPc molecule increased as the energy difference between the downshifted graphene EF and the lowest unoccupied molecular orbital (LUMO) coincided with the excitation laser energy, which is in accordance with the CM [50]. The authors concluded that the band alignment between the cobalt-Pc (CoPc) molecule and graphene was responsible for the changes of the Raman intensities of the molecules on graphene.

Moreover, another intensively used strategy to change the electronic properties of 2D materials is to introduce surface defects to the 2D materials, which consequently changes the band alignment between the molecule and 2D materials. In 2011, Huh et al. first showed that by simply exposing graphene to UV/ozone treatment, the intensity of the 1648 cm^−1^ mode of absorbed rhodamine B (RhB) was much stronger than that on an as-prepared sample [46]. An EF of ~10^4^ (Figure 3c) was reported on this UV/ozone treated graphene, which was more than two orders of magnitude larger than that on pristine graphene. In this work, the influence of UV/ozone treatment to the Raman enhancement was attributed to the following aspects: (1) Oxygen functional groups induced by the treatment result in a larger polarizability of the surface, which eventually enhanced the interaction between the molecule and the surface; (2) the functionalization downshifted the E_F_ of graphene, resulting in a p-type dope in graphene and, consequently, a better energy level alignment for the CM. Later, in 2014, Sun et al. showed that the Raman enhancement effect on MoS_2_ nanoflakes could also benefit from a similar plasma treatment [51]. It was found that R6G molecules deposited on plasma treated MoS_2_ displayed a reduced photoluminescence background and more resolvable Raman bands. More recently, in 2018, Liu et al. reported that introducing Se vacancies to CVD grown WSe_2_ could largely enhance the Raman signals of CuPc [47]. When the atomic ratio of Se and W in WSe_2_ decreased from 2 to 1.96, the EF for the 1528 cm^−1^ mode of CuPc increased by over 40 times when compared to the as-prepared sample (Figure 3d), thus leading to an enhancement of 126 times compared to CuPc molecules deposited on quartz substrates.

**Figure 4:**
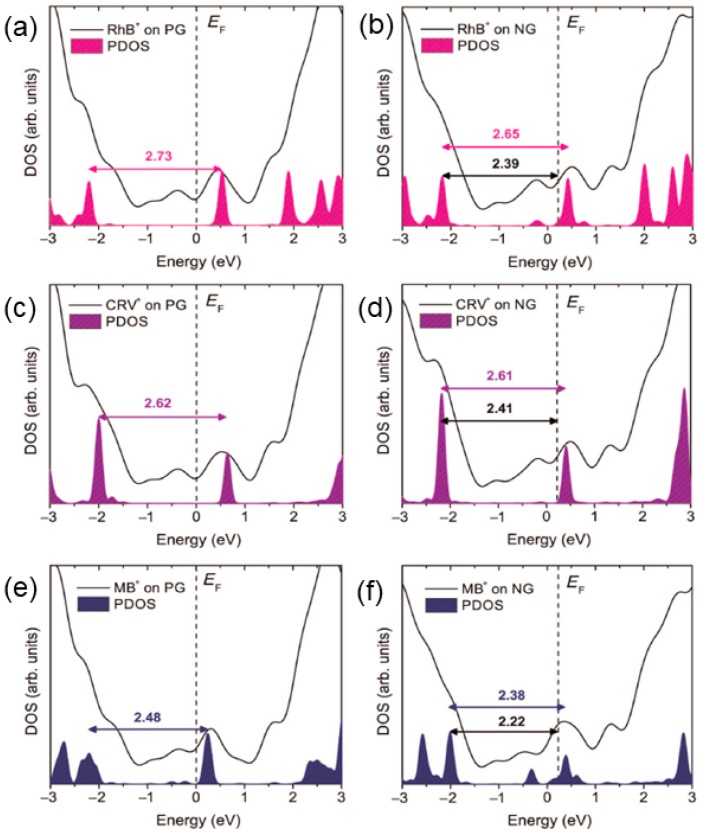
Densities of states (DOS) of the clusters representing the adsorbed organic dyes on PG and NG. (**a**,**b**) for rhodamine B (RhB); (**c**,**d**) for crystal violet (CRV), and (**e**,**f**) for methylene blue (MB). The filled areas are the densities of states (PDOS) projected on the dyes. Vertical dashed lines indicate the position of the system’s Fermi energy, E_F_. Horizontal arrows and values indicate the HOMO-LUMO gaps in electronvolt (eV), and the energy, HOMO-E_F_, in eV [18].

N-doped graphene monolayers have also been used as GERS substrates to probe various dye molecules, and extraordinary molecular sensing properties were observed when compared to pristine graphene. For example, the Raman signal of rhodamine B can be detected for concentrations as low as 10^−11^ mol/L, which is the lowest ever reported value when using graphene as a substrate so far [18]. With this concentration, the number of molecules within the laser spot is on the magnitude of 10^1^, close to single molecule detection [18]. Electronic structure calculations and simulations of the Raman spectra by density functional theory (DFT) suggest that charge transfer is the mechanism for GERS. The Raman enhancement of the molecules studied is more significant for N-doped graphene (NG) than it is for pristine graphene (PG) since the LUMO levels of the dyes are closer to the Fermi level of NG (Figure 4). There has been no correlation between the dye molecule symmetry and the enhancement of the Raman signal, according to the analysis of the data obtained. Furthermore, the resonant Raman condition can be achieved by using an excitation energy equal to the energy gap between the highest occupied molecular orbital (HOMO) and the lowest unoccupied molecular orbital (LUMO) of the molecule, which improves the sensitivity of GERS detection.

### 2.3. Dependence on the Molecular Configuration

**Figure 5:**
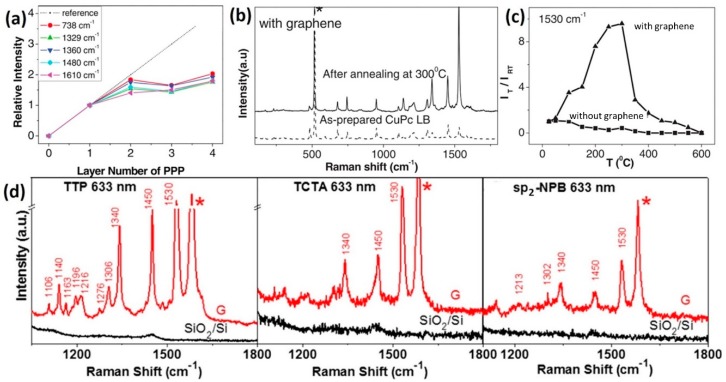
(**a**) First-layer effect in GERS of PPP molecules deposited using an LB technique. Reproduced from [55] with permission (Wiley-VCH, 2010). (**b**) Enhanced Raman scattering of CuPc on graphene after annealing treatment due to the change of the molecular orientation. (**c**) Intensity change of the 1530 cm^−1^ mode as a function of the annealing temperature. Reproduced from [56] with permission (Wiley-VCH, 2012). (**d**) GERS effect for tetrathienophenazine (TTP), tris(4-carbazoyl-9-ylphenyl) amine (TCTA), and 2,2′7,7′-tetra(N-phenyl-1-naphthyl-amine)-9,9′-spirobifluorene (sp_2_-NPB) molecules. Reproduced from [57] with permission (American Chemical Society, 2015).

Studies on the molecular configuration dependent GERS further provide strong evidence to support the CM mechanism in GERS [52]. In the CM description, it is very important that the molecules are adsorbed well on the substrate surface so that charge-transfers and vibronic couplings can occur. Therefore, the first-layer effect, which states that only the first layer of molecules closest to the surface can be enhanced through a CM [53,54], should be present in GERS. Indeed, this effect has been confirmed by studying the dependence of the GERS enhancement effect on the number of molecular layers. Different layers of PPP molecules were deposited on graphene via a Langmuir-Blodgett (LB) technique, in which single layers of ordered PPP can be sequentially added to the substrate in a controlled manner [55]. Figure 5a shows the intensity of the selected modes as a function of the number of layers of PPP molecules, indicating that the Raman intensity does not increase much after the first layer deposition. It provides strong evidence that the first layer of PPP molecules contributes the most to the enhancement, in accordance with the short-range nature of the CM.

Besides the need for direct contact between the molecule and the substrate, the molecular orientation was also found to play an important role in GERS. Ling et al. constructed “upstanding” CuPc molecules on graphene, and changed them to a “lying down” configuration through an annealing step [56]. When comparing the Raman spectra of these two scenarios, it was found that the enhancement is stronger when the CuPc molecules are lying down, due to enhanced π-π interactions established between CuPc and graphene (Figure 5b). In particular, the intensities of the CuPc Raman signals (e.g., 1530 cm^−1^ mode) reached a maximum when annealing at around 300 °C, when all the molecules are lying down over the entire surface. It was also noticed that the intensity of the sample annealed at 600 °C was comparable to the as-prepared LB film (i.e., molecules are upstanding), even though fewer molecules are present due to desorption of the molecules during the annealing process (Figure 5c).

Proper quantitative analysis requires both the enhanced Raman signals on the 2D materials and the unenhanced signals on the bare substrate to be observed. For this reason, the probe molecules used in GERS studies have large Raman scattering cross sections (e.g., Pc molecules). This leads to a fundamental question about which molecules are better suitable for detection via GERS. Huang et al. performed a systematic study on the molecular selectivity of GERS, in which various molecules with similar structures, but distinct energy level alignment with respect to graphene (e.g., metal-Pc), or with similar energy level alignment, but distinct chemical structures, were used [57]. For example, tetrathienophenazine (TTP), tris(4-carbazoyl-9-ylphenyl) amine (TCTA), and 2,2′7,7′-tetra(N-phenyl-1-naphthyl-amine)-9,9′-spirobifluorene (sp_2_-NPB), all have similar energy levels when compared to Pc, but very distinct structures belonging to the D_2h_, C_3_, and S_4_ symmetries, respectively. The highest EF for the modes located at ~1450 cm^−1^ was 23.3 for TTP, 6.9 for TCTA, and 4.3 for sp_2_-NPB, when compared to the substrate (Figure 5d). This suggests that, in addition to appropriate energy level alignments, molecules with a suitable structure and symmetry benefit the most in the enhancement effect, because they can have greater overlaps with the graphene’s electron cloud, being in close contact with graphene.

### 2.4. Quantum Description of the CM in Molecule/2D Material Systems

**Figure 6:**
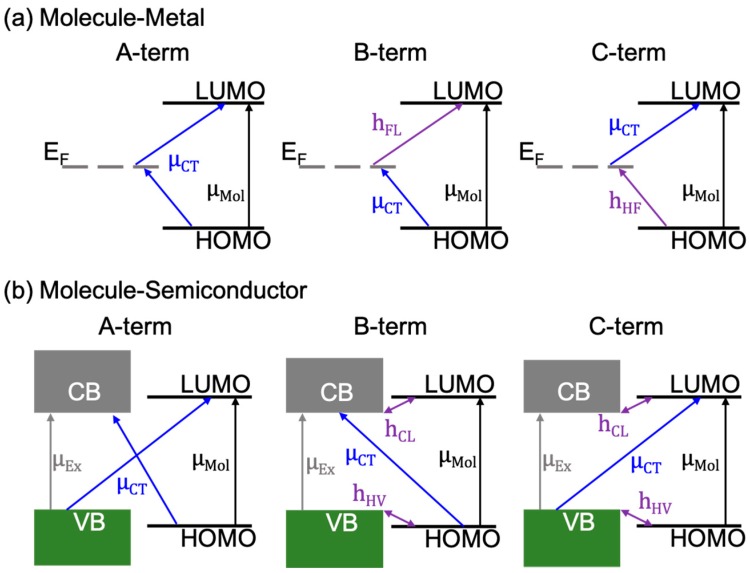
Possible resonances in the chemical mechanism (CM) for a (**a**) molecule/metal, and (**b**) molecule/semiconductor system.

Based on the Raman intensity theory of SERS, where the polarizability (*α*) of the molecule can be described as a sum of three terms, A, B, and C [58], a quantum description of CM was widely applied to the molecule/2D materials system in the literature. The A-term describes intensities stemming from resonances in the molecule-2D material system, including molecular, excitonic, and charge-transfer (CT) resonances. The B- and C-terms account for CT resonances enabled by intensity “borrowing” (through vibronic couplings) of allowed molecular or excitonic resonances, respectively. For example, for a molecule/metallic 2D material (e.g., graphene) system, charge transfers in the A-term can occur from the HOMO of the molecule to the E_F_ of the 2D material or from the E_F_ to the LUMO (Figure 6a). The charge-transfers enabled by the B-term originate from the HOMO of the molecule to the E_F_ of the 2D material, assisted by a vibronic coupling of the E_F_ of the 2D material and the LUMO of the molecule. The case responsible for the C-term is very similar, but the charge-transfer occurs between the E_F_ and the LUMO, and the vibronic coupling occurs between the E_F_ and the HOMO.

With the opening of a bandgap in 2D semiconductors, such as MoS_2_ and WSe_2_, the A-, B-, and C-terms undergo slight modifications. In addition to a new excitonic resonance in the semiconducting 2D materials, the CT resonances in the A-term can occur from the HOMO of the molecule to the conduction band (CB) of the semiconductor, or from the valence band (VB) of the semiconductor to the LUMO of the molecule. Charge-transfers in the B-term occur from the HOMO to the CB through intensity borrowing of an excitonic resonance (μ_Ex_: Transition dipole moment of the excitonic resonance), which is made possible by the vibronic coupling of the HOMO and the VB (h_HV_). The CT contributing to the B-term can also occur from borrowing the intensity of a molecular resonance (μ_Mol_: Transition dipole moment of the molecular resonance), by the vibrionic coupling between LUMO and CB (h_CL_). The CT contribution to the C-term is very similar to the B-term, but with the CT between the VB of the 2D semiconductor and the LUMO of the molecule. The vibronic couplings and excitonic and molecular resonances responsible for the C-term are the same as that for the B-term, as shown in Figure 6b. In particular, the charge-transfer dipole moment (μ_CT_) for B- and C-terms will be large when the vibronic coupling is strong. Moreover, as all resonances and couplings are linked [59,60], large signal enhancements should be expected when the laser excitation energy matches one or more of the following: (1) μ_CT_; (2) μ_Mol_; and; (3) μ_Ex_ [61].

**Figure 7:**
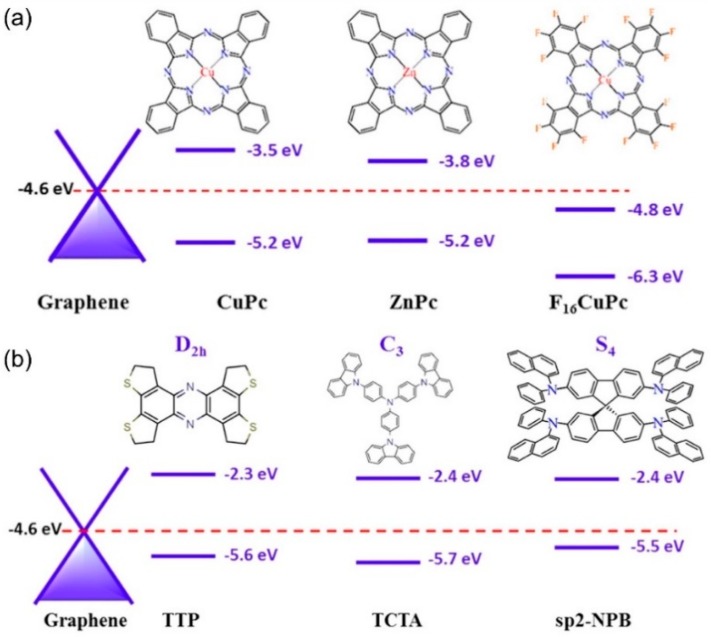
Energy band alignment between graphene’s Fermi level and several molecules with (**a**) similar symmetries, but differing HOMO-LUMO positions (CuPc, ZnPc, F_16_CuPc), and (**b**) different symmetries with similar HOMO-LUMO positions (TTP, TCTA, sp_2_-NPB) [57].

As the field makes progress in elucidating the Raman enhancement mechanism when using different 2D systems as the SERS substrate, it should be noted that while a qualitative analysis is possible with the current theoretical framework, a quantitative model is still lacking. From the discussion above, it can be rationalized that the energy band alignment between the molecule and the substrate is key. In fact, an energy band alignment analysis may help explain why CuPc has larger signal enhancements than other molecules with similar symmetries, such as ZnPc and F_16_CuPc (Figure 7a). However, the energy band alignment alone is not sufficient to determine the level of enhancement, as molecules with similar HOMO-LUMO positions (TTT, TCTA, and sp_2_-NPB, Figure 7b) are not equally amplified, and other factors may be at play [57]. Future directions should expand to include other 2D materials that offer larger breadth in optical and electronic properties, so that the chemical mechanism can be studied in better detail. In addition, from the experimental design, molecular deposition methods with better control of the uniformity and the number of molecules, such as vacuum evaporation and Langmuir-Blodgett techniques, could be used to provide quantitative and reliable enhancement factor data for the development of a theoretical model. As such, Table 1 compares several molecule/2D material systems according to their EF, detection limit, deposition method, and probe molecule. Overall, we realize that 2D materials constitute a novel platform for a deep understanding of the CM and are becoming more prevalent in enhanced Raman spectroscopy due to their atomically flat nature and tunable properties. It is worth mentioning that a vertical graphene/WSe_2_ heterostructure was reported recently to achieve a larger EF than the sum of their individual counterparts, thus indicating that the interlayer coupling also plays a key role when tuning the properties of substrates and influencing the enhancement effects [33]. The diverse possible combinations of the 2D materials also open a broader avenue for both the CM study and potential applications.

## 3. Applications of GERS in Chemical and Biological Sensing

### 3.1. Chemical Sensing

In general, an ideal sensor must be sensitive, specific, and minimally perturbative to the target analyte: It cannot be sensitive to anything that is not the target analyte, and it must measure the analyte without changing the property to be measured. Conventional SERS systems have struggled with the latter, as the metal surface required for plasmonic enhancement is chemically active, and minimizes reproducibility [67]. As graphene is inert in comparison, GERS would be ideal for sensing; however, there are still challenges to overcome. The selectivity/specificity of GERS needs to be improved especially because the Raman modes of graphene fall in the same spectral ranges as many organic molecules. GERS also has lower enhancement, ranging from 10 to 100 times [32,68] the intensity of conventional Raman, when compared to factors of 10^4^–10^9^ for plasmonic SERS [69,70,71]. However, recent advances in GERS sensing have begun to address these challenges [57].

**Figure 8:**
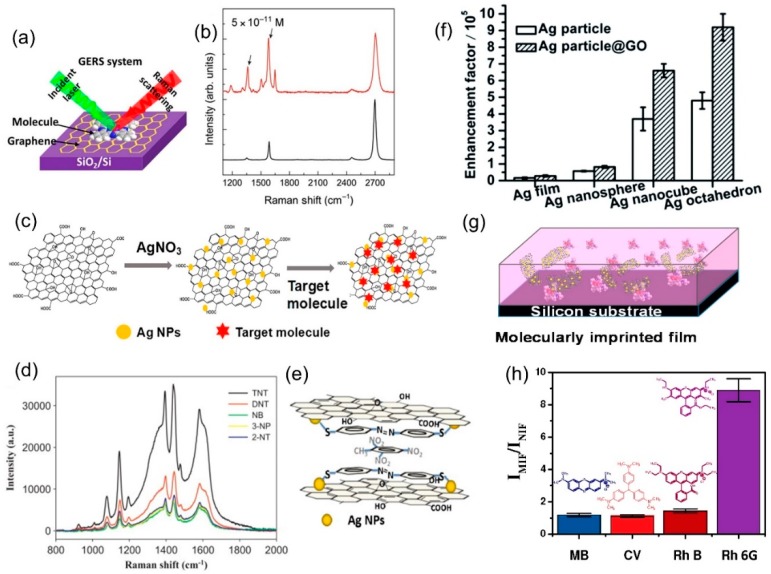
(**a**) Schematic of the GERS system. Reproduced from [67] with permission (American Chemical Society, 2015). (**b**) Raman of rhodamine B on pristine graphene (bottom) and n-doped graphene (top), showing a detectable limit of 10^−11^ M [18]. (**c**) Schematic of the one-pot synthesis of graphene/Ag nanoparticle (G/AgNP) composite used for graphene-mediated surface enhanced Raman scattering (G-SERS). Reproduced from [74] with permission (Elsevier, 2018). (**d**) Raman spectra of p-aminothiophenol (PATP)-functionalized G/AgNPs with 2,4,6-trinitrotoluene (TNT)-like structures at the same concentration, showing selectivity. (**e**) Scheme of the proposed enhancement mechanism for PATP-modified G/AgNPs detecting TNT. Reproduced from [75] with permission (Elsevier, 2013). (**f**) Enhancement factor of different shapes of Ag nanoparticles with and without graphene oxide (GO). Reproduced from [76] with permission (Royal Society of Chemistry, 2012). (**g**) Schematic of the molecularly imprinted (MI) GO/AgNP porous structure [77]. (**h**) Selectivity of the MI structure, showing the EF of several structural analogs of Rh6G (the template molecule). The EF is shown relative to the intensity observed for a non-imprinted GO/AgNP porous structure [77].

The Raman enhancement of graphene is mainly attributed to the CM [57,72], among which the formation of π-π bonds can improve charge transfer between graphene and the adsorbed molecules (Figure 8a). It is therefore logical to assume that modifying the electronic states of graphene would affect the enhancement. This has been demonstrated by Xu et al. with a graphene field-effect transistor device [45]. By changing the Fermi level of graphene via an applied voltage, the enhancement factor for dye molecules can be strengthened or weakened. Further testing under different atmospheres revealed that graphene is effectively doped by gas species adsorbed onto the surface, which combined with the Fermi level shift from the applied field, results in a hysteresis effect under different biases [73]. Although the non-linearity of the effect is not ideal for sensing, it demonstrates the possibility of tuning the alignment of the Fermi level of graphene and the LUMO of an analyte molecule to maximize enhancement [57]. Indeed, chemical doping of graphene has been used by Feng et al. to align the graphene Fermi level with the LUMO of fluorescent dye molecules, greatly improving the enhancement factor and allowing detection of concentrations as low as 10^−11^ M (Figure 8b), the lowest reported for GERS [18].

An additional method of enhancing selectivity is via molecular imprinting. A molecularly imprinting polymer matrix is created by the self-assembly of monomers around a template molecule (the target analyte); once the template is removed, a cavity is left and it will selectively fit the template structure. The inclusion of graphene nanosheets in the polymer matrix can provide enhancement, resulting in a hybrid nanocomposite that is highly stable [74], physically robust, and capable of highly selective molecular sensing [78]. Carboni et al. first demonstrated this approach with a porous-silica based nanocomposite with an EF of 14.64 [79]. Furthermore, the nanocomposite showed a 4.5 times stronger enhancement specifically for its template molecule (Rh6G), compared to the EF for a structurally similar dye molecule (RhB). Most recently, they have demonstrated the use of this structure to detect the organic pollutant, Paraoxon, in water with the same degree of selectivity and sensitivity [79].

Graphene nanocomposites can also be used to improve the EF and selectivity, specifically the combination of graphene with noble metal nanoparticles. This combines the stronger plasmonic-based EM of metals with the superior stability, adsorption, and quenching of graphene [80]. The inclusion of the noble metal nanoparticles also leads to preservation of the desired roughness, a decrease in the amount of material used (and therefore cost), and the prevention of graphene aggregation during processing [78]. This hybrid graphene-mediated surface enhanced Raman scattering (G-SERS) method has been widely applied for chemical sensors. A general scheme for graphene/silver nanoparticle (NP) composite is shown in Figure 8c. In this context, Xie et al. reported a graphene/silver (G/Ag) nanocomposite, synthesized by one-pot synthesis of Ag nanoparticles on graphene oxide (GO) with sodium citrate reduction [80]. The G/Ag nanocomposite was used to identify synthetic food dyes based on their Raman spectra. The dyes were detected at concentrations as low as 10^−7^ M, and individual dyes could be identified from their characteristic peaks in a mixture of four dyes. Liu and Chen reported a similar silver/graphene nanosheet (Ag/GNs) composite, used to detect 2,4,6-trinitrotoluene (TNT) [75]. The nanosheets were further functionalized with p-aminothiophenol (PATP) to improve selectivity. The interaction of PATP and TNT creates a complex on top of the Ag/GNs, effectively creating multiple layers of π-π conjugated structures on top of graphene, thus massively increasing the CM enhancement on top of the EM of the Ag nanoparticles. This resulted in the detection of concentrations as low as 10^−11^ M. Furthermore, the EF of TNT was around two to four times higher when compared to similar compounds, such as 2,4-dinitrotoluene, which did not create π-π stacking with PATP (Figure 8d,e).

Further refinements of the G/Ag system includes the work of Li et al., who used G/Ag to selectively detect polar antibiotic compounds in water [81]; Fan et al., who demonstrated the tuning of EF by changing the shape of the nanoparticles [76] (Figure 8f); and Liu et al., who showed increased sensitivity by using Ag nanorods to monitor the concentration of iodine in solutions, all of which enable rapid and facile detection of the target analyte at concentrations of 10^−8^ M, 10^−9^ M, and 10^−10^ M, respectively [82]. Beyond Ag nanoparticles, Carboni et al. have proposed a nanocomposite made of graphene nanosheets dispersed in mesoporous titania, or Ti-GERS [83]. Though the exact enhancement mechanism for this nanocomposite has not yet been elucidated, it is capable of achieving an analytical EF of 16 times when compared to normal Raman scattering. Another approach, reported by Zhang et al., combines silver-coated gold nanoparticles (Au@Ag NPs) with GO in a layered sandwich structure for in-situ detection of the pesticide thiram, down to 10^−7^ M concentrations [84]. In addition, Jiang et al. have improved the molecular imprinting method by combining the polymer matrix with dispersed GO/Ag nanosheets (Figure 8g), and achieved an EF of 712 with respect to Raman scattering without enhancement, and an impressive selectivity, with Rh6G having an EF around 8 times larger than rhodamine B [77] (Figure 8h).

### 3.2. Biomolecule Sensing

Biosensing is an important sub-field of sensing, with applications in drug discovery, diagnosis, food safety, and security [85]. When compared to chemical sensing, biosensing introduces additional requirements on the sensor. It requires extreme sensitivity as target analytes can be present in very small concentrations. For example, early detection of cancer biomarkers requires sensitivity at concentrations as low as 10^−12^ M to ensure an accurate diagnosis [86]. Selectivity is also difficult, as the sensing device can quickly become fouled with non-target molecules in complex biological media. The sensor should also be biocompatible, which rules out the use of toxic or highly reactive species that can disrupt biological processes. SERS in general already has major advantages in biosensing, as it potentially combines single-molecule sensitivity, molecular fingerprinting, and rapid measurements. GERS, in particular, has the added bonus of the facile tuning of surface properties, which can be leveraged to selectively adsorb the target analytes [87]. Graphene is also not considered to be toxic, although questions regarding the effects of factors, like surface chemistry and dosage, on long-term health remain unanswered [88].

**Figure 9:**
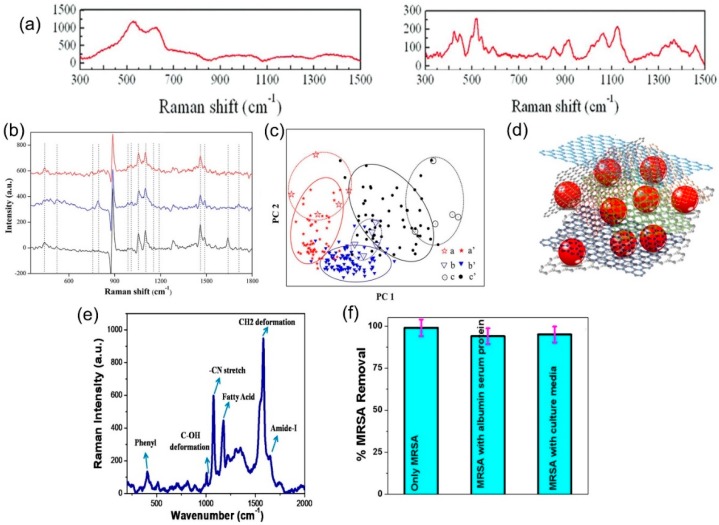
(**a**) Raman spectra of 0.55 mM glucose on copper substrate (left) and on as-grown graphene on copper (right). Reproduced from [91] with permission (Royal Society of Chemistry, 2015). (**b**) Raman spectra of 14 gastric cancer biomarkers (characteristic peaks marked by dotted lines) in real/simulated breath samples from patients with advanced gastric cancer (top), early gastric cancer (middle), and healthy individuals (bottom). (**c**) Principal component analysis of the spectra in (**b**), showing how healthy samples are distinguished from early or advanced cancer samples. Reproduced from [96] with permission (American Chemical Society, 2016). (**d**) Schematic of the chitosan—GO/AuNP porous membrane described in [97]. The Au NPs (shown in red) are wrapped in chitosan chains and mixed with GO before being cross-linked into the final membrane. (**e**) Raman spectra of drug-resistant bacteria methicillin-resistant *Staphylococcus aureus* (MRSA) using the multifunctional porous membrane. (**f**) Percent removal of MRSA dispersed in water, a solution of other proteins, and a solution containing culture media. Reproduced from [97] with permission (American Chemical Society, 2017).

GERS is useful for the detection of biomarkers, which can be cells, molecules, proteins, or enzymes, and is used to detect or monitor a disease state, drug response, or normal biological processes. For example, Huang et al. used GERS to detect blood constituent proteins, including hemoglobin and albumin, which are vital indicators of health problems, such as leukemia and lung diseases [89]. Another common example is the detection of blood glucose levels, which is vital for patients with diabetes. Measuring blood glucose, optically, is attractive because it is non-invasive, rapid, and easy. Table 2 shows a comparison of glucose detection methods, including a commercial meter, graphene-based electrochemical sensor, SERS with Ag nanospheres (measured in vivo), and the various GERS-based devices discussed in this review. Gupta et al. demonstrated an Ag@AuNP/GO nanocomposite as a glucose biosensor [90]. With the nanoparticles functionalized by mercaptophenyl boronic acid, a linear response range of 2.0–6.0 × 10^−3^ M was achieved in human blood samples, even when using a test solution containing additional proteins. The work of Chattopadhy et al. shows that as-grown graphene can be used to detect glucose in a buffer solution with impressive sensitivity, with a linear range of 10–500 mg/dL (0.55–27.75 × 10^−3^ M) [91]. Figure 9a shows a comparison of the glucose Raman spectra collected with and without GERS. More recently, an improved G-SERS device was reported by Li et al. based on GO/gold nanorods (AuNRs), which measured the change in the Raman activity of oxidized ascorbic acid in response to the glucose concentration [92]. The measured peak intensity is linearly and inversely proportional to the concentration of glucose in water between 10^−4^–10^−1^ M, even when mixed with other common components of blood. Overall, GERS offers the possibility of sensitive and accurate determination of blood glucose levels, which can be collected non-invasively and continuously. There is still room for improvement regarding the detection limit to rival other existing technologies, such as electrochemical and photoelectrical methods.

Although glucose is one of the simplest and most-studied biomarkers, GERS can be used for more complicated ones. For example, Xu et al. have presented a direct-growth single-step G/CuNP nanocomposite, which can detect adenosine, a key molecule in metabolic processes, nerve impulse propagation, and a precursor to DNA and RNA [98]. The G/CuNPs enabled the detection of adenosine linearly from 5–500 × 10^−9^ M, and its characteristic peaks could be detected in human urine samples, though its reproducibility is somewhat lacking.

Moreover, biomarker detection with Raman spectroscopy can be multiplexed, meaning it is not limited to a single species at a time. In this context, Chen et al. have demonstrated simultaneous detection of 14 biomarkers associated with gastric cancer using a GO/AuNP nanocomposite for G-SERS [96]. Using the variations in the Raman intensity of the 14 biomarkers (shown in Figure 9b), it was possible to distinguish healthy patients from those with early gastric cancer from those with advanced gastric cancer, using only a breath sample adsorbed onto the G-SERS sensor. Figure 9c shows the principal component analysis mapping of the samples; despite variations in the 14 biomarkers, the healthy, early, and advanced samples cluster into distinguishable groups. G-SERS can also be used for multiplex detection of DNA. For example, He et al. presented an improvement over conventional fluorescence methods, using a G/AuNP nanocomposite for easy detection of multiple DNA samples [99]. The AuNPs were functionalized via capture DNA to bind the target DNA, and an EF of 1.8 × 10^4^ was observed, with the capture DNA enabling high specificity as well.

Using a similar sensor, Fan et al. demonstrated label-free detection of HIV DNA; the unique graphene/gold nano-popcorn structure enabled an EF of 3.8 × 10^11^, with the extra enhancement being attributed to the higher roughness (thus an increased number of hotspots) of the nano-popcorn shape [100]. When used to detect a characteristic sequence of HIV DNA, the authors showed very high sensitivity with detection limits down to 10^−15^ M, and high reproducibility. Manikandan et al. have also used G-SERS to detect cancer cells via G/AuNPs, and have shown a 5-fold increase in sensitivity, which could lead to the early detection of cancer cells [101]. Most impressively, Jones et al. have created a multifunctional nanocomposite-embedded porous membrane, which can adsorb contaminants from water (thus purifying water) while simultaneously providing a G-SERS platform for identification of the contaminants [97]. The approach involves a combination of chitosan (an antimicrobial biopolymer) on AuNPs attached to graphene, which enables the detection of antibiotics at concentrations as low as 10^−10^ M, while also removing around 85% of the selected drugs spiked in river water. Figure 9d,e shows the schematic of the nanocomposite, and Raman spectra of antibiotic-resistant bacteria. The membrane was also tested with antibiotic-resistant bacteria with a removal efficiency of close to 100% (Figure 9f), thus demonstrating a low-cost scalable multifunctional porous membrane that can filter and identify water-based biological contaminants.

## 4. Conclusions

In conclusion, GERS is an emerging method for chemical- and bio-sensing, with the advantages of high signal reproducibility, uniformity, and compatibility with biosystems. The full utilization of GERS as a sensing method still requires ongoing efforts. First, the synthesis of graphene and the related 2D materials, as well as the precise control of the doping level and Fermi energy, will be a basis to achieve sensitive, and controllable GERS sensors. The fundamental principles of GERS have been investigated, yet more in-depth and thorough surveys of the types of molecules need to be considered, to fully understand the GERS mechanisms. In addition, the deployment of GERS in various chemical and biological settings still requires different methods to modify and improve the enhancement substrate, to achieve highly competitive sensing performance for a wide variety of applications.

## Figures and Tables

**Table 1:** EFs and detection limits for diverse molecule/2D material systems.

Reference	Substrate	Molecule ^a^	Deposition Method ^b^	Detection Limit (mol/L)	Enhancement Factor
[32]	Graphene	Pc	TE	N/A	2–17
		R6G, PPP	SS	8 × 10^−10^ M (R6G) 2 × 10^−8^ M (PPP)	N/A
[46]	Graphene oxide	R6G	SS	N/A	1 × 10^4^
[62]	Reduced graphene oxide	RhB	SS	5 × 10^−8^ M	1 × 10^3^
[63]	Graphene quantum dots	R6G	DC	1 × 10^−9^ M	2.37 × 10^3^
[18]	N-doped graphene	RhB	SS	5 × 10^−11^ M	N/A
[47]	Defective WSe_2_	CuPc	LB film	N/A	126
[64]	MoS_2_	4-Mpy	DC	N/A	3.8 × 10^5^
[65]	Oxidized MoS_2_	R6G	SS	1 × 10^−7^ M	1.4 × 10^5^
[66]	WTe_2_	R6G	DC	4 × 10^−14^ M	1.8 × 10^9^
	MoTe_2_	R6G	DC	4 × 10^−13^ M	1.6 × 10^8^

(**a**) Pc: Phthalocyanine, R6G: Rhodamine 6G, PPP: Porphyrin, RhB: Rhodamine B, CuPc: Copper phthalocyanine, 4-Mpy: 4-mercaptopyridine. (**b**) TE: Thermal evaporation, SS: Solution soaking, DC: Drop casting, LB film: Langmuir-Blodgett film.

**Table 2:** Comparison of glucose sensing methods, detection limit, and detection linear range.

Method	Detection Limit (mM)	Detection Linear Range (mM)	Reference
Commercial meter	0.6	1.1–33.3	[93]
Electrochemical—graphene electrode	0.035	0.1–10	[94]
SERS	-	5.55–27.75	[95]
G-SERS	0.33	2.0–6.0	[90]
G-SERS	0.1	0.1–100	[92]
GERS	0.55	0.55–27.75	[91]
